# Dyspnea in a case of shoulder dislocation – to beware of this rare life-threatening symptom

**DOI:** 10.1051/sicotj/2016022

**Published:** 2016-09-23

**Authors:** Satyen Praful Joshi, Nikhil Subhash Challawar, Parth Vinod Agrawal, Arpit S. Gajjar

**Affiliations:** 1 Dr. Vasantrao Pawar Medical College & Research Center Nashik Maharashtra 422003 India

**Keywords:** Shoulder dislocation, Dyspnea, Intra-thoracic migration of head of humerus

## Abstract

Shoulder dislocation is a common injury in orthopedic practice. In an acute presentation, closed reduction of the shoulder joint leads to an uneventful recovery. However, in the developing world neglected shoulder dislocation and treatments from quacks are not uncommon. Improper treatment and neglect can rarely become life threatening. We present one such case, emphasizing the need to investigate the symptom of dyspnea in a patient with history of shoulder dislocation.

## Case

A 68-year-old diabetic had a fall down a staircase, following which he had severe pain and restriction of movement at his right shoulder joint. He went to a quack who manipulated his shoulder. The pain decreased significantly with some improvement manipulated in his global range of motion at the shoulder joint.

**Figure 1.**
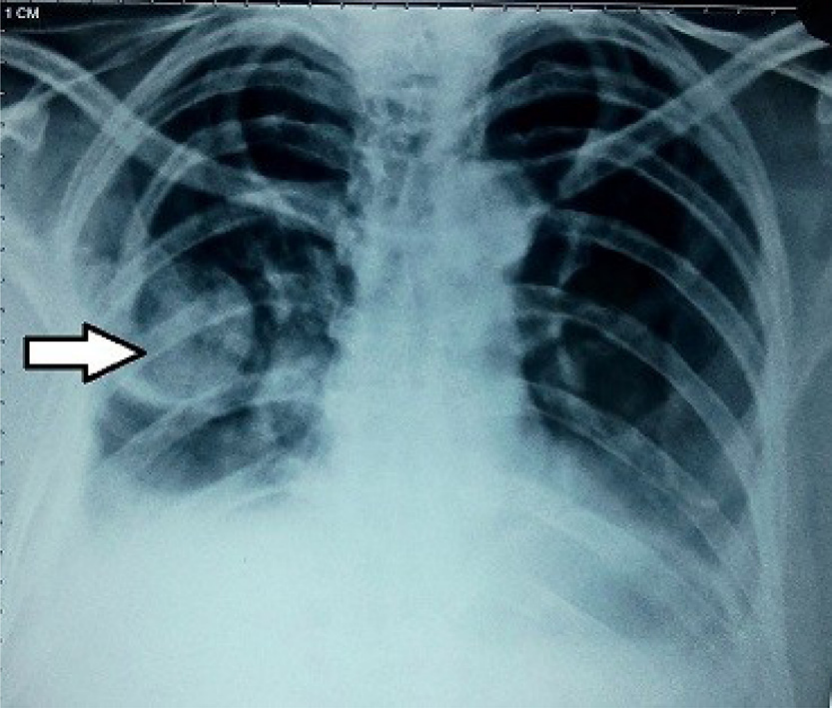
X-ray showing a well defined circular opacity in middle zone of right lung.

**Figure 2.**
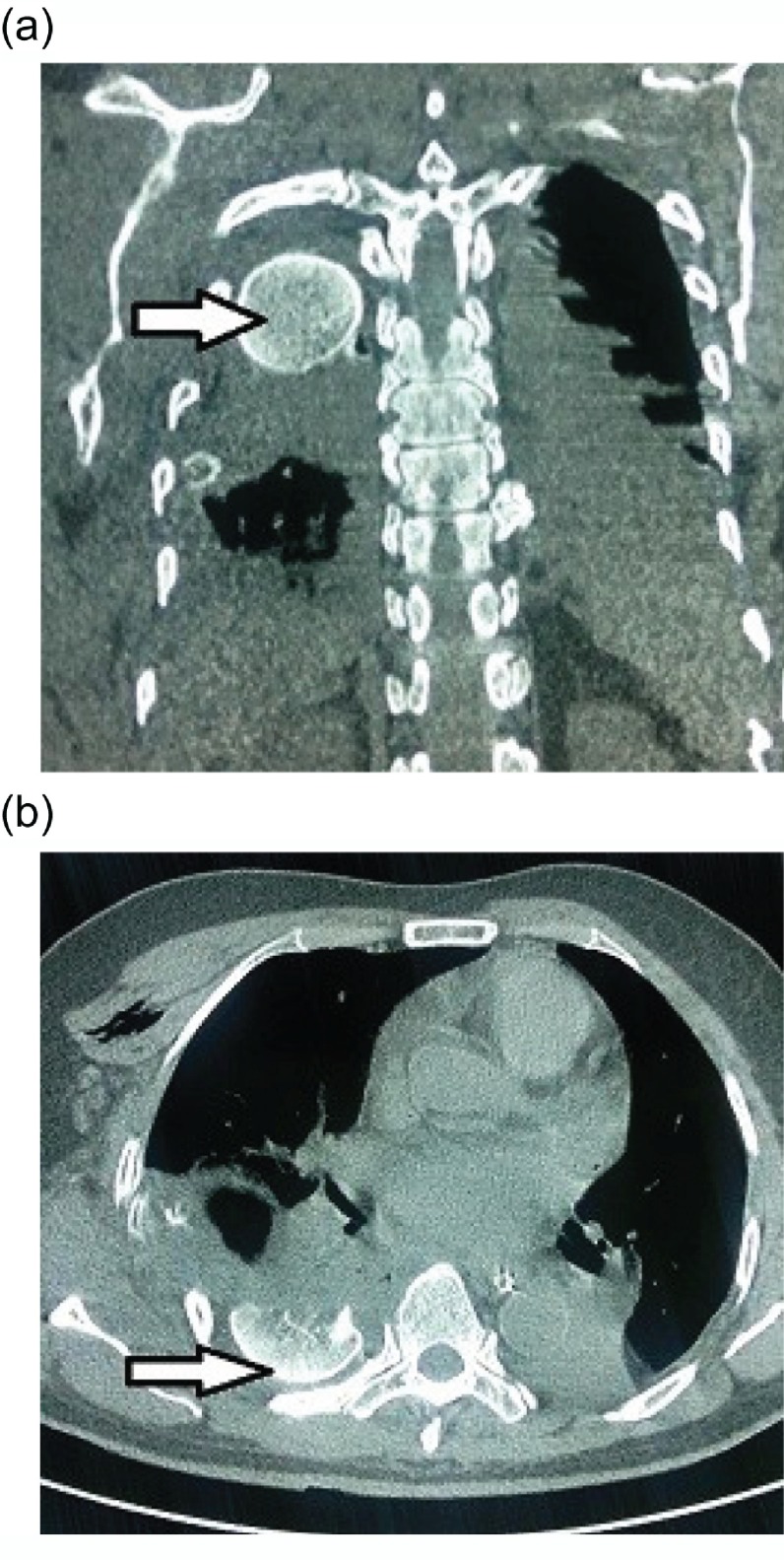
(a) Coronal plane CT showing intra-thoracic humeral head on right side. (b) Axial plane CT showing pleural effusion with right sided intrathoracic humeral head.

**Figure 3.**
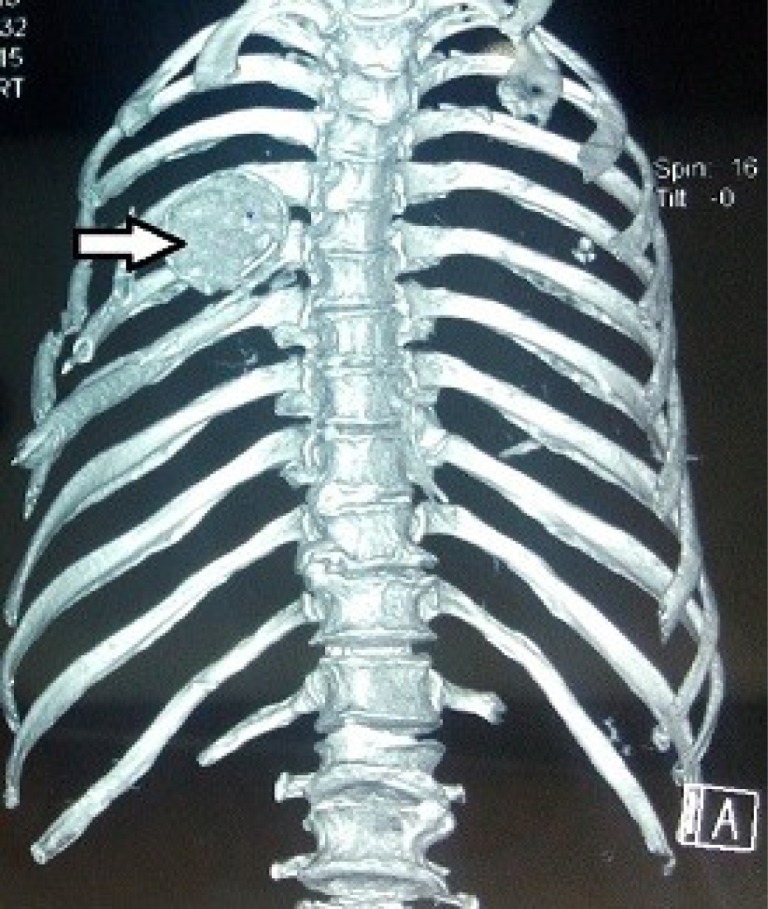
3D reconstruction CT showing right sided intrathoracic humeral head with fractured ribs on ipsilateral side.

However, after four days the patient started complaining of dyspnea. The dyspnea was aggravated by exertion and activity, but also persisted at rest. Preliminary evaluation revealed an opaque patch in the midzone of right thorax on chest X-ray (Figure 1). Part of the humeral head was missing from the glenoid. A CT scan confirmed that the fractured humeral head had migrated into the right thoracic cavity (Figures 2 and 3). Overlying ribs on the ipsilateral side were also fractured together with hemothorax.

An intercostal drainage tube was inserted to drain the hemothorax, following which an open thoracotomy was performed to remove the humeral head. A Neer’s prosthesis was inserted through deltopectoral approach to regain functional mobility of the right shoulder joint.

Dyspnea subsided within a week after the surgery. At a two-year follow-up the patient has abduction to 120° and is able to carry out his low demand daily activities with ease.

## Discussion

Fracture dislocation of proximal humerus with intrathoracic migration of the head is extremely rare [1]. In our case a violent unscientific maneuver probably caused levering of the humeral head over the 2nd to 5th ribs causing rib fractures as well as the intrathoracic migration of the humeral head. It is probable that the intra-thoracic fracture dislocation occurred at the time of injury. The Quack may have completed the fracture allowing the shaft to return extra-thoracic. This caused partial improvement of movement at the shoulder joint, but caused rest pain in the right chest along with dyspnea.

Excision of the humeral head has to be done immediately to prevent further complications like pneumonitis, visceral impaction and acute respiratory distress syndrome (ARDS) [2]. In the absence of intra-thoracic complications, the removal of humeral head may not be necessary [2]. There is no uniform guideline for the treatment of this injury and each case must be appropriately managed according to its specific features [3]. A post reduction X-ray of a shoulder dislocation ensuring correct placement of humeral head in glenoid cavity will help in avoiding such complications.

The humeral head replacement with a prosthesis remains an issue of debate among orthopedic surgeons. Excision arthroplasty at the shoulder joint can improve the range of motion but can impair the quality of life with decreased strength and pain at shoulder joint [1]. Hence we preferred to replace the humeral head, though greater tuberosity reconstruction was not possible in this case.

## Conclusion

Dyspnea in a case of shoulder dislocation is a very rare symptom. Though rare, the treating physician should promptly evaluate this symptom. A migrated humeral head may cause life-threatening symptoms and urgent removal of the head is necessary.

## Conflict of interest

The authors declare no conflict of interest in relation with this paper.
